# Supplementary material for: Dietary Patterns, Serum BDNF and Fatty Acid Profiles in Physically Active Male Young Adults: A Cluster Analysis Study
Source: Nutrients. 2024 Dec 15;16(24):4326. doi: 10.3390/nu16244326 (PMC11679842; doi:10.3390/nu16244326)
Supplement: Supplementary file 1 [file nutrients-16-04326-s001.zip › nutrients-3324420-supplementary.pdf]

nutrients

MDPI

Table S1. Frequency of consumption of different types of food.

food frequency consumption		never	1-3 times a month	once a week	few times a week	once a day	few times a day
white bread and bakery products	n	8	15	16	68	43	22
	%	4.7	8.7	9.3	39.5	25.0	12.8
wholemeal (brown) bread/bread rolls	n	20	44	29	64	8	7
	%	11.6	25.6	16.9	37.2	4.7	4.1
white rice, white pasta, fine-ground groats, e.g. semolina, couscous	n	3	19	24	100	21	5
	%	1.7	11.0	14.0	58.1	12.2	2.9
buckwheat, oats, wholegrain pasta or other coarse-ground groats	n	10	50	38	60	12	2
	%	5.8	29.1	22.1	34.9	7.0	1.2
fast foods	n	8	80	58	24	1	1
	%	4.7	46.5	33.7	14.0	0.6	0.6
fried foods	n	2	12	20	114	19	5
	%	1.2	7.0	11.6	66.3	11.0	2.9
butter	n	27	17	21	64	32	11
	%	15.7	9.9	12.2	37.2	18.6	6.4
lard	n	130	30	8	4	0	0
	%	75.6	17.4	4.7	2.3	0.0	0.0
vegetable oils or margarines or mixes of butter and margarines	n	71	34	20	33	11	3
	%	41.3	19.8	11.6	19.2	6.4	1.7
milk	n	7	33	28	57	32	15
	%	4.1	19.2	16.3	33.1	18.6	8.7
fermented milk beverages	n	14	30	32	63	24	9
	%	8.1	17.4	18.6	36.6	14.0	5.2
fresh cheese curd products	n	17	45	45	46	18	1
	%	9.9	26.2	26.2	26.7	10.5	0.6
cheese	n	6	22	35	79	23	7
	%	3.5	12.8	20.3	45.9	13.4	4.1
cold meats, smoked sausages, hot-dogs	n	4	19	26	78	34	11
	%	2.3	11.0	15.1	45.3	19.8	6.4
red meat	n	10	48	49	56	7	2
	%	5.8	27.9	28.5	32.6	4.1	1.2
white meat	n	2	7	21	117	21	4
	%	1.2	4.1	12.2	68.0	12.2	2.3
fish	n	19	84	62	5	2	0
	%	11.0	48.8	36.0	2.9	1.2	0.0
eggs	n	3	23	30	82	30	4
	%	1.7	13.4	17.4	47.7	17.4	2.3
pulses-based foods	n	24	86	43	17	2	0
	%	14.0	50.0	25.0	9.9	1.2	0.0
potatoes	n	5	41	53	69	4	0
	%	2.9	23.8	30.8	40.1	2.3	0.0
fruit	n	2	11	25	67	43	24
	%	1.2	6.4	14.5	39.0	25.0	14.0
vegetables	n	1	8	17	64	39	43
	%	0.6	4.7	9.9	37.2	22.7	25.0

sweets	n	6	22	37	70	20	17
	%	3.5	12.8	21.5	40.7	11.6	9.9
instant soups or ready-made soups	n	103	55	11	2	1	0
	%	59.9	32.0	6.4	1.2	0.6	0.0
tinned (jar) meats	n	118	44	7	3	0	0
	%	68.6	25.6	4.1	1.7	0.0	0.0
tinned (jar) vegetables	n	70	57	24	16	4	1
	%	40.7	33.1	14.0	9.3	2.3	0.6
fruit juices	n	16	57	41	44	8	6
	%	9.3	33.1	23.8	25.6	4.7	3.5
vegetable juices or fruit and vegetable juices	n	56	76	18	18	3	1
	%	32.6	44.2	10.5	10.5	1.7	0.6
sweetened hot beverages, such as black tea, coffee, herbal or fruit teas	n	40	24	21	34	21	32
	%	23.3	14.0	12.2	19.8	12.2	18.6
sweetened carbonated or still beverages	n	30	61	33	35	6	7
	%	17.4	35.5	19.2	20.3	3.5	4.1
energy drinks	n	59	44	35	24	6	4
	%	34.3	25.6	20.3	14.0	3.5	2.3
water	n	3	3	5	26	12	123
	%	1.7	1.7	2.9	15.1	7.0	71.5
alcoholic beverages	n	30	87	40	14	0	1

Table S2. Classification results of LDA presenting percentage of predicted cluster membership for actual cluster.

	Predicted clusters membership			
**Actual cluster**	**Correct classification**	**Cl1**	**Cl2**	**Cl3**
Cl1	86.96%	20	2	1
Cl2	88.89%	0	56	7
Cl3	91.86%	1	6	79
**Total**	**90.12%**	**21**	**64**	**87**

Table S3. Characteristic of revealed clusters.

		**Cl1**	**Cl2**	**Cl3**	**p value**
age		21.7 ± 2.2	22.2 ± 3.0	21.7 ± 2.4	0.4575
hight[cm]		184 ± 8	180 ± 15	180 ± 13	0.5630
body mass [kg]		81 ± 11	80 ± 10	81 ± 11	0.6011
BMI		24.0 ± 2.6	24.0 ± 2.2	24.7 ± 2.9	0.1976
fat [%]		13.2 ± 3.8	13.1 ± 3.7	13.9 ± 3.9	0.4355
fat mass [kg]		11.0 ± 4.3	10.7 ± 4.1	11.6 ± 4.9	0.4259
fat-free mass [kg]		70.1 ± 7.2	69.0 ± 7.4	69.9 ± 7.4	0.7429
total body water [kg]		46.8 ± 5.7	46.1 ± 5.7	46.9 ± 5.8	0.7335
place of residence	Village	3 13.0%	7 11.1%	17 19.8%	0.2789
	Town below 20.000 inhabitants	0 0.0%	4 6.3%	6 7.0%	
	Town between 20.000 and 100.000 inhabitants	4 17.4%	18 28.6%	13 15.1%	
	City over 100.000 inhabitants	16 69.6%	34 54.0%	50 58.1%	
financial situation	Below average	0 0.0%	9 14.3%	6 7.0%	0.1972
	Average	19 82.6%	40 63.5%	58 67.4%	
	Above average	4 17.4%	14 22.2%	22 25.6%	

work status	No, I am retired or receiving a disability living allowance	0	0.0%	0	0.0%	2	2.3%	0.4029
	No, I am on maternity leave	0	0.0%	0	0.0%	0	0.0%	
	Yes, but it is only a temporary job	11	47.8%	27	42.9%	42	48.8%	
	Yes, I am permanently employed	6	26.1%	9	14.3%	17	19.8%	
	No, I study	6	26.1%	27	42.9%	25	29.1%	
education	Primary	0	0.0%	1	1.6%	4	4.7%	0.4958
	Lower secondary	1	4.3%	1	1.6%	0	0.0%	
	Upper secondary	14	60.9%	36	57.1%	52	60.5%	
	Higher (e.g. BSc, MSc)	8	34.8%	25	39.7%	30	34.9%	
following a diet	No	19	82.6%	58	92.1%	72	83.7%	0.2787
	Yes, as advised by my doctor for medical reasons	0	0.0%	0	0.0%	0	0.0%	
	Yes, it was my personal decision	4	17.4%	5	7.9%	14	16.3%	
physical activity at work or school	Low	3	13.0%	11	17.5%	18	20.9%	0.8055
	Moderate	9	39.1%	25	39.7%	37	43.0%	
	High	11	47.8%	27	42.9%	31	36.0%	
physical activity during time off	Low	1	4.3%	4	6.3%	5	5.8%	0.8931
	Moderate	5	21.7%	20	31.7%	24	27.9%	
	High	17	73.9%	39	61.9%	57	66.3%	
integrated physical activity level	Low	1	4.3%	7	11.1%	14	16.3%	0.5494
	Moderate	13	56.5%	37	58.7%	48	55.8%	
	High	9	39.1%	19	30.2%	24	27.9%	
tobacco smoking	No	21	91.3%	54	85.7%	73	84.9%	0.7290
	Yes	2	8.7%	9	14.3%	13	15.1%	
tobacco smoking in the pass	No	13	56.5%	38	60.3%	56	65.1%	0.6972
	Yes	10	43.5%	25	39.7%	30	34.9%	
hours of sleep (weekdays)	≤6 hours/day	8	34.8%	23	36.5%	23	26.7%	0.6350
	6-9 hours/day	15	65.2%	38	60.3%	61	70.9%	
	> 9 hours/day	0	0.0%	2	3.2%	2	2.3%	
hours of sleep (weekends)	≤6 hours/day	2	8.7%	4	6.3%	4	4.7%	0.3630
	6-9 hours/day	14	60.9%	34	54.0%	60	69.8%	
	> 9 hours/day	7	30.4%	25	39.7%	22	25.6%	
health status	Worse than others	2	8.7%	4	6.3%	9	10.5%	0.2081
	The same as others	5	21.7%	27	42.9%	23	26.7%	
	Better than others	16	69.6%	32	50.8%	54	62.8%	
nutrition knowledge (self-evaluation)	Insufficient	2	8.7%	13	20.6%	9	10.5%	0.0564
	Sufficient	5	21.7%	27	42.9%	28	32.6%	
	Good	13	56.5%	17	27.0%	43	50.0%	
	Very good	3	13.0%	6	9.5%	6	7.0%	
nutrition knowledge	Insufficient	1	4.3%	15	23.8%	11	12.8%	0.1281
	Sufficient	16	69.6%	40	63.5%	61	70.9%	
	Good	6	26.1%	8	12.7%	14	16.3%	
diet (self-evaluation)	Very bad	1	4.3%	1	1.6%	2	2.3%	0.8969
	Bad	6	26.1%	16	25.4%	16	18.6%	
	Good	15	65.2%	44	69.8%	63	73.3%	
	Very good	1	4.3%	2	3.2%	5	5.8%	

weekdays to weekends diet comparison	No difference really	11	47.8%	29	46.0%	43	50.0%	0.9744
	Differs slightly	10	43.5%	27	42.9%	36	41.9%	
	Very different	2	8.7%	7	11.1%	7	8.1%	

Table S4. Frequency of consumption of different types of food in revealed clusters.

		Cl1	Cl2	Cl3	p value
white bread and bakery products	Never	2	2	4	0.61938
		8.70%	3.17%	4.65%	
	1-3 times a month	3	2	10	
		13.04%	3.17%	11.63%	
	Once a week	2	5	9	
		8.70%	7.94%	10.47%	
	Few times a week	9	25	34	
		39.13%	39.68%	39.53%	
	Once a day	5	21	17	
		21.74%	33.33%	19.77%	
	Few times a day	2	8	12	
		8.70%	12.70%	13.95%	
wholemeal (brown) bread/bread rolls	Never	1	10	9	0.00138
		4.35%	15.87%	10.47%	
	1-3 times a month	1	25	18	
		4.35%	39.68%	20.93%	
	Once a week	3	13	13	
		13.04%	20.63%	15.12%	
	Few times a week	14	14	36	
		60.87%	22.22%	41.86%	
	Once a day	3	0	5	
		13.04%	0.00%	5.81%	
	Few times a day	1	1	5	
		4.35%	1.59%	5.81%	
white rice, white pasta, fine-ground groats	Never	0	0	3	0.15119
		0.00%	0.00%	3.49%	
	1-3 times a month	2	9	8	
		8.70%	14.29%	9.30%	
	Once a week	0	9	15	
		0.00%	14.29%	17.44%	
	Few times a week	15	38	47	
		65.22%	60.32%	54.65%	
	Once a day	6	6	9	
		26.09%	9.52%	10.47%	
	Few times a day	0	1	4	
		0.00%	1.59%	4.65%	
buckwheat, oats, wholegrain pasta or other coarse-ground groats	Never	0	6	4	0.00516
		0.00%	9.52%	4.65%	
	1-3 times a month	0	22	28	
		0.00%	34.92%	32.56%	
	Once a week	4	15	19	
		17.39%	23.81%	22.09%	
	Few times a week	15	18	27	
		65.22%	28.57%	31.40%	

	Once a day	4	2	6	
		17.39%	3.17%	6.98%	
	Few times a day	0	0	2	
		0.00%	0.00%	2.33%	
fast foods	Never	0	1	7	0.26723
		0.00%	1.59%	8.14%	
	1-3 times a month	11	24	45	
		47.83%	38.10%	52.33%	
	Once a week	9	25	24	
		39.13%	39.68%	27.91%	
	Few times a week	3	11	10	
		13.04%	17.46%	11.63%	
	Once a day	0	1	0	
		0.00%	1.59%	0.00%	
	Few times a day	0	1	0	
		0.00%	1.59%	0.00%	
fried foods	Never	0	1	1	0.14146
		0.00%	1.59%	1.16%	
	1-3 times a month	0	5	7	
		0.00%	7.94%	8.14%	
	Once a week	1	6	13	
		4.35%	9.52%	15.12%	
	Few times a week	20	47	47	
		86.96%	74.60%	54.65%	
	Once a day	2	3	14	
		8.70%	4.76%	16.28%	
	Few times a day	0	1	4	
		0.00%	1.59%	4.65%	
butter	Never	5	2	20	0.01736
		21.74%	3.17%	23.26%	
	1-3 times a month	0	5	12	
		0.00%	7.94%	13.95%	
	Once a week	2	7	12	
		8.70%	11.11%	13.95%	
	Few times a week	8	30	26	
		34.78%	47.62%	30.23%	
	Once a day	7	14	11	
		30.43%	22.22%	12.79%	
	Few times a day	1	5	5	
		4.35%	7.94%	5.81%	
lard	Never	14	48	68	0.23512
		60.87%	76.19%	79.07%	
	1-3 times a month	8	9	13	
		34.78%	14.29%	15.12%	
	Once a week	1	3	4	
		4.35%	4.76%	4.65%	
	Few times a week	0	3	1	
		0.00%	4.76%	1.16%	
	Once a day	0	0	0	
		0.00%	0.00%	0.00%	
	Few times a day	0	0	0	

		0.00%	0.00%	0.00%	
vegetable oils or margarines or mixes of butter and margarines	Never	10	19	42	0.08953
		43.48%	30.16%	48.84%	
	1-3 times a month	6	15	13	
		26.09%	23.81%	15.12%	
	Once a week	0	11	9	
		0.00%	17.46%	10.47%	
	Few times a week	4	16	13	
		17.39%	25.40%	15.12%	
	Once a day	2	1	8	
		8.70%	1.59%	9.30%	
	Few times a day	1	1	1	
		4.35%	1.59%	1.16%	
milk	Never	0	2	5	0.00008
		0.00%	3.17%	5.81%	
	1-3 times a month	1	13	19	
		4.35%	20.63%	22.09%	
	Once a week	0	17	11	
		0.00%	26.98%	12.79%	
	Few times a week	6	22	29	
		26.09%	34.92%	33.72%	
	Once a day	9	7	16	
		39.13%	11.11%	18.60%	
	Few times a day	7	2	6	
		30.43%	3.17%	6.98%	
fermented milk beverages	Never	0	6	8	0.00117
		0.00%	9.52%	9.30%	
	1-3 times a month	0	13	17	
		0.00%	20.63%	19.77%	
	Once a week	1	19	12	
		4.35%	30.16%	13.95%	
	Few times a week	12	20	31	
		52.17%	31.75%	36.05%	
	Once a day	8	4	12	
		34.78%	6.35%	13.95%	
	Few times a day	2	1	6	
		8.70%	1.59%	6.98%	
fresh cheese curd products	Never	0	7	10	0.03656
		0.00%	11.11%	11.63%	
	1-3 times a month	2	18	25	
		8.70%	28.57%	29.07%	
	Once a week	5	18	22	
		21.74%	28.57%	25.58%	
	Few times a week	10	18	18	
		43.48%	28.57%	20.93%	
	Once a day	6	2	10	
		26.09%	3.17%	11.63%	
	Few times a day	0	0	1	
		0.00%	0.00%	1.16%	
cheese	Never	0	1	5	0.18055
		0.00%	1.59%	5.81%	

	1-3 times a month	2	8	12	
		8.70%	12.70%	13.95%	
	Once a week	1	12	22	
		4.35%	19.05%	25.58%	
	Few times a week	16	28	35	
		69.57%	44.44%	40.70%	
	Once a day	4	11	8	
		17.39%	17.46%	9.30%	
	Few times a day	0	3	4	
		0.00%	4.76%	4.65%	
cold meats, smoked sausages, hot-dogs	Never	2	0	2	0.20488
		8.70%	0.00%	2.33%	
	1-3 times a month	3	6	10	
		13.04%	9.52%	11.63%	
	Once a week	4	9	13	
		17.39%	14.29%	15.12%	
	Few times a week	12	31	35	
		52.17%	49.21%	40.70%	
	Once a day	2	15	17	
		8.70%	23.81%	19.77%	
	Few times a day	0	2	9	
		0.00%	3.17%	10.47%	
red meat,	Never	2	1	7	0.33272
		8.70%	1.59%	8.14%	
	1-3 times a month	6	16	26	
		26.09%	25.40%	30.23%	
	Once a week	7	21	21	
		30.43%	33.33%	24.42%	
	Few times a week	8	24	24	
		34.78%	38.10%	27.91%	
	Once a day	0	1	6	
		0.00%	1.59%	6.98%	
	Few times a day	0	0	2	
		0.00%	0.00%	2.33%	
white meat	Never	1	0	1	0.24241
		4.35%	0.00%	1.16%	
	1-3 times a month	2	2	3	
		8.70%	3.17%	3.49%	
	Once a week	0	11	10	
		0.00%	17.46%	11.63%	
	Few times a week	19	42	56	
		82.61%	66.67%	65.12%	
	Once a day	1	6	14	
		4.35%	9.52%	16.28%	
	Few times a day	0	2	2	
		0.00%	3.17%	2.33%	
fish	Never	1	8	10	0.01881
		4.35%	12.70%	11.63%	
	1-3 times a month	6	28	50	
		26.09%	44.44%	58.14%	
	Once a week	13	25	24	

		56.52%	39.68%	27.91%	
	Few times a week	3	1	1	
		13.04%	1.59%	1.16%	
	Once a day	0	1	1	
		0.00%	1.59%	1.16%	
	Few times a day	0	0	0	
		0.00%	0.00%	0.00%	
eggs	Never	1	1	1	
		4.35%	1.59%	1.16%	
	1-3 times a month	1	10	12	
		4.35%	15.87%	13.95%	
	Once a week	2	17	11	
		8.70%	26.98%	12.79%	
	Few times a week	17	28	37	
		73.91%	44.44%	43.02%	
	Once a day	1	6	23	
		4.35%	9.52%	26.74%	
	Few times a day	1	1	2	
		4.35%	1.59%	2.33%	
pulses-based foods	Never	2	12	10	0.00472
		8.70%	19.05%	11.63%	
	1-3 times a month	5	34	47	
		21.74%	53.97%	54.65%	
	Once a week	9	14	20	
		39.13%	22.22%	23.26%	
	Few times a week	7	3	7	
		30.43%	4.76%	8.14%	
	Once a day	0	0	2	
		0.00%	0.00%	2.33%	
	Few times a day	0	0	0	
		0.00%	0.00%	0.00%	
potatoes	Never	2	2	1	0.03512
		8.70%	3.17%	1.16%	
	1-3 times a month	4	8	29	
		17.39%	12.70%	33.72%	
	Once a week	4	24	25	
		17.39%	38.10%	29.07%	
	Few times a week	13	27	29	
		56.52%	42.86%	33.72%	
	Once a day	0	2	2	
		0.00%	3.17%	2.33%	
	Few times a day	0	0	0	
		0.00%	0.00%	0.00%	
fruit	Never	0	1	1	0.00315
		0.00%	1.59%	1.16%	
	1-3 times a month	0	5	6	
		0.00%	7.94%	6.98%	
	Once a week	0	9	16	
		0.00%	14.29%	18.60%	
	Few times a week	5	31	31	
		21.74%	49.21%	36.05%	

	Once a day	9<br>39.13%	14<br>22.22%	20<br>23.26%	
	Few times a day	9<br>39.13%	3<br>4.76%	12<br>13.95%	
vegetables	Never	0<br>0.00%	1<br>1.59%	0<br>0.00%	0.00205
	1-3 times a month	0<br>0.00%	3<br>4.76%	5<br>5.81%	
	Once a week	0<br>0.00%	10<br>15.87%	7<br>8.14%	
	Few times a week	3<br>13.04%	29<br>46.03%	32<br>37.21%	
	Once a day	7<br>30.43%	13<br>20.63%	19<br>22.09%	
	Few times a day	13<br>56.52%	7<br>11.11%	23<br>26.74%	
sweets	Never	0<br>0.00%	0<br>0.00%	6<br>6.98%	0.02666
	1-3 times a month	0<br>0.00%	4<br>6.35%	18<br>20.93%	
	Once a week	4<br>17.39%	15<br>23.81%	18<br>20.93%	
	Few times a week	13<br>56.52%	27<br>42.86%	30<br>34.88%	
	Once a day	3<br>13.04%	9<br>14.29%	8<br>9.30%	
	Few times a day	3<br>13.04%	8<br>12.70%	6<br>6.98%	
instant soups or ready-made soups	Never	10<br>43.48%	33<br>52.38%	60<br>69.77%	0.00063
	1-3 times a month	13<br>56.52%	17<br>26.98%	25<br>29.07%	
	Once a week	0<br>0.00%	10<br>15.87%	1<br>1.16%	
	Few times a week	0<br>0.00%	2<br>3.17%	0<br>0.00%	
	Once a day	0<br>0.00%	1<br>1.59%	0<br>0.00%	
	Few times a day	0<br>0.00%	0<br>0.00%	0<br>0.00%	
tinned (jar) meats	Never	13<br>56.52%	42<br>66.67%	63<br>73.26%	0.34847
	1-3 times a month	9<br>39.13%	18<br>28.57%	17<br>19.77%	
	Once a week	1<br>4.35%	1<br>1.59%	5<br>5.81%	
	Few times a week	0<br>0.00%	2<br>3.17%	1<br>1.16%	
	Once a day	0<br>0.00%	0<br>0.00%	0<br>0.00%	
	Few times a day	0	0	0	

		0.00%	0.00%	0.00%	
tinned (jar) vegetables	Never	8<br>34.78%	34<br>53.97%	28<br>32.56%	0.01422
	1-3 times a month	7<br>30.43%	18<br>28.57%	32<br>37.21%	
	Once a week	5<br>21.74%	10<br>15.87%	9<br>10.47%	
	Few times a week	3<br>13.04%	0<br>0.00%	13<br>15.12%	
	Once a day	0<br>0.00%	0<br>0.00%	4<br>4.65%	
	Few times a day	0<br>0.00%	1<br>1.59%	0<br>0.00%	
fruit juices	Never	0<br>0.00%	1<br>1.59%	15<br>17.44%	<0.00001
	1-3 times a month	2<br>8.70%	14<br>22.22%	41<br>47.67%	
	Once a week	7<br>30.43%	16<br>25.40%	18<br>20.93%	
	Few times a week	10<br>43.48%	22<br>34.92%	12<br>13.95%	
	Once a day	4<br>17.39%	4<br>6.35%	0<br>0.00%	
	Few times a day	0<br>0.00%	6<br>9.52%	0<br>0.00%	
vegetable or fruit and vegetable juices	Never	1<br>4.35%	21<br>33.33%	34<br>39.53%	0.00005
	1-3 times a month	9<br>39.13%	24<br>38.10%	43<br>50.00%	
	Once a week	3<br>13.04%	8<br>12.70%	7<br>8.14%	
	Few times a week	8<br>34.78%	8<br>12.70%	2<br>2.33%	
	Once a day	2<br>8.70%	1<br>1.59%	0<br>0.00%	
	Few times a day	0<br>0.00%	1<br>1.59%	0<br>0.00%	
sweetened hot beverages	Never	1<br>4.35%	0<br>0.00%	39<br>45.35%	<0.00001
	1-3 times a month	0<br>0.00%	7<br>11.11%	17<br>19.77%	
	Once a week	1<br>4.35%	15<br>23.81%	5<br>5.81%	
	Few times a week	7<br>30.43%	17<br>26.98%	10<br>11.63%	
	Once a day	4<br>17.39%	10<br>15.87%	7<br>8.14%	
	Few times a day	10<br>43.48%	14<br>22.22%	8<br>9.30%	
sweetened carbonated or still beverages	Never	3<br>13.04%	5<br>7.94%	22<br>25.58%	0.00006

	1-3 times a month	5	17	39	
		21.74%	26.98%	45.35%	
	Once a week	4	15	14	
		17.39%	23.81%	16.28%	
	Few times a week	9	15	11	
		39.13%	23.81%	12.79%	
	Once a day	0	6	0	
		0.00%	9.52%	0.00%	
	Few times a day	2	5	0	
		8.70%	7.94%	0.00%	
energy drinks	Never	4	17	38	0.00551
		17.39%	26.98%	44.19%	
	1-3 times a month	3	15	26	
		13.04%	23.81%	30.23%	
	Once a week	7	18	10	
		30.43%	28.57%	11.63%	
	Few times a week	8	9	7	
		34.78%	14.29%	8.14%	
	Once a day	0	3	3	
		0.00%	4.76%	3.49%	
	Few times a day	1	1	2	
		4.35%	1.59%	2.33%	
water	Never	0	1	2	0.90256
		0.00%	1.59%	2.33%	
	1-3 times a month	0	2	1	
		0.00%	3.17%	1.16%	
	Once a week	1	2	2	
		4.35%	3.17%	2.33%	
	Few times a week	4	10	12	
		17.39%	15.87%	13.95%	
	Once a day	0	4	8	
		0.00%	6.35%	9.30%	
	Few times a day	18	44	61	
		78.26%	69.84%	70.93%	
alcoholic beverages	Never	4	5	21	0.11544
		17.39%	7.94%	24.42%	
	1-3 times a month	9	34	44	
		39.13%	53.97%	51.16%	
	Once a week	6	17	17	
		26.09%	26.98%	19.77%	
	Few times a week	4	6	4	
		17.39%	9.52%	4.65%	
	Once a day	0	0	0	
		0.00%	0.00%	0.00%	
	Few times a day	0	1	0	
		0.00%	1.59%	0.00%	

Table S5. Values of fatty acid indices in serum of individuals of revealed clusters**.**

	**Cl1**	**Cl2**	**Cl3**	**p value**
A-SFA	309 ± 248[a]	285 ± 266	195 ± 137[a]	0.0142
T-SFA	387 ± 311[a]	365 ± 340	250 ± 179[a]	0.0157
A-SFA/FA total	0.247 ± 0.020	0.238 ± 0.026	0.237 ± 0.023	n.s.
T-SFA/FA total	0.311 ± 0.021	0.305 ± 0.030	0.304 ± 0.028	n.s.
$^{A\text{-}SFA}$ index	0.437 ± 0.060	0.415 ± 0.092	0.412 ± 0.076	n.s.
$^{T\text{-}SFA}$ index	351 ± 309	332 ± 307	233 ± 164	n.s.
SFA/UFA	0.496 ± 0.052	0.485 ± 0.079	0.483 ± 0.073	n.s.
SFA/PUFA	0.84 ± 0.14	0.81 ± 0.18	0.78 ± 0.16	n.s.
SFA/MUFA	1.26 ± 0.19	1.25 ± 0.24	1.33 ± 0.38	n.s.
SFA/FA	0.330 ± 0.023	0.324 ± 0.033	0.323 ± 0.031	n.s.
MUFA/FA	0.267 ± 0.039	0.266 ± 0.042	0.252 ± 0.039	n.s.
PUFA/FA	0.401 ± 0.048	0.408 ± 0.050	0.423 ± 0.045	n.s.
PUFA/SFA	1.23 ± 0.21	1.28 ± 0.24	1.32 ± 0.22	n.s.
UFA/SFA	2.04 ± 0.22	2.11 ± 0.30	2.11 ± 0.30	n.s.
n6/n3 PUFA	12.8 ± 3.6	13.0 ± 3.0	13.2 ± 3.4	n.s.
h/H Ch	2.47 ± 0.31	2.60 ± 0.43	2.62 ± 0.37	n.s.
$^{C18:0}\Delta 9$ index	0.760 ± 0.037	0.73 ± 0.14	0.731 ± 0.074	n.s.
$^{C16:0}\Delta 9$ index	0.074 ± 0.024[a]	0.064 ± 0.019	0.058 ± 0.017[a]	0.0036
$^{C14:0}\Delta 9$ index	0.125 ± 0.034	0.130 ± 0.039	0.13 ± 0.10	n.s.
$^{t11C18:1}\Delta 9$ index	0.43 ± 0.26	0.45 ± 0.22	0.40 ± 0.19	n.s.
$^{\Sigma}\Delta 9$ index	0.428 ± 0.042	0.420 ± 0.082	0.410 ± 0.066	n.s.
$^{\Sigma\Delta 9,6,5,4}$ index	0.691 ± 0.021	0.696 ± 0.029	0.697 ± 0.029	n.s.
$^{n6elongC20/C18}$ index	0.0043 ± 0.0011	0.0047 ± 0.0013	0.0043 ± 0.0013	n.s.
$^{n6elongC22/C20}$ index	0.41 ± 0.10	0.440 ± 0.094	0.416 ± 0.089	n.s.
$^{\Delta 4desaturation}$index	0.744 ± 0.059	0.744 ± 0.066	0.751 ± 0.068	n.s.
$^{\Delta 5desaturation}$index	0.9730 ± 0.0084[a]	0.975 ± 0.011	0.9780 ± 0.0096[a]	0.0174

Data are shown as mean values ± standard deviation (SD). P value ≤0.05—significant differences among groups in Kruskal-Wallis test. Values sharing a letter in one row are significantly different ($p < 0.05$) in multiple comparison test.

A-SFA= C12:0 + C14:0 + C16:0; T-SFA = C14:0 + C16:0 + C18:0; $^{A\text{-}SFA}$index= (C12:0 + 4 × C14:0 + C16:0) / (ΣMUFA + Σn-6PUFA + Σn-3PUFA); $^{T\text{-}SFA}$index = (C14:0 + C16:0 + C18:0) / [(0.5 × ΣMUFA + 0.5 × Σn-6PUFA + 3 × Σn-3PUFA) / Σn-6PUFA)]; $^{C18:0\Delta 9}$index = c9C18:1/(c9C18:1+C18:0); $^{\Sigma\Delta 9,6,5,4FA}$index = (ΣMUFA+ΣPUFA)/(C16:0+C18:0+C20:0+C22:0+C24:0 +ΣMUFA+ΣPUFA); $^{n\text{-}6ElongC20/C18index}$ = c11c14C20:2/(c11c14C20:2+LA); $^{n\text{-}3ElongC22/C20}$index = DPA/(DPA+EPA);$^{\Delta 4desaturation}$index = DHA/(DHA+DPA); $^{\Delta 5desaturation}$index = AA/(AA+c8c11c14C20:3); h/H-Ch = (c7C18:1+c9C18:1+c12C18:1+c14C18:1+c11C20:1+13C22:1+LA+ALA+c6c9c12C18:3+AA+c11c14C20:2+EPA+c7c10c13c16C22:4+ DPA)/(C14:0+C16:0); FA – fatty acids; A-SFA-atherogenic saturated fatty acids; T-thrombogenic saturated fatty acids; h/H-Ch – ratio of hipo- to hipercholesterolemic fatty acids; SFA- saturated fatty acids; UFA – unsaturated fatty acids; MUFA – monounsaturated fatty acids; PUFA – polyunsaturated fatty acids; LA – linoleic acid (c9c12C18:2); DPA – docosapentaenoic acid (c7c10c13c16c19C22:5); DHA – docosahexaenoic acid (c4c7c10c13c16c19C22:6); EPA – eicosapentaenoic acid (c5c8c11c14c17C20:5); AA – arachidonic acid (c5c8c11c14C20:4).
